# Prehospital resuscitation with hypertonic saline-dextran modulates inflammatory, coagulation and endothelial activation marker profiles in severe traumatic brain injured patients

**DOI:** 10.1186/1742-2094-7-5

**Published:** 2010-01-18

**Authors:** Shawn G Rhind, Naomi T Crnko, Andrew J Baker, Laurie J Morrison, Pang N Shek, Sandro Scarpelini, Sandro B Rizoli

**Affiliations:** 1 Defence Research and Development Canada (DRDC), Toronto, Canada; 2 Graduate Program in Kinesiology and Health Science, York University, Toronto, Canada; 3 Department of Laboratory Medicine and Pathobiology, Faculty of Medicine, University of Toronto, Canada; 4 Brain Injury Laboratory, Cara Phelan Centre for Trauma Research Keenan Research Centre, Li Ka Shing Knowledge Institute, St Michael's Hospital, University of Toronto, Toronto, Ontario, Canada; 5 Critical Care Medicine, St Michael's Hospital, University of Toronto, Ontario, Canada; 6 Rescu, Keenan Research Centre, Li Ka Shing Knowledge Institute, St Michael's Hospital, University of Toronto, Toronto, Ontario, Canada; 7 Department of Surgery and Critical Care Medicine, Sunnybrook Health Sciences Centre, Toronto, Ontario, Canada

## Abstract

**Background:**

Traumatic brain injury (TBI) initiates interrelated inflammatory and coagulation cascades characterized by wide-spread cellular activation, induction of leukocyte and endothelial cell adhesion molecules and release of soluble pro/antiinflammatory cytokines and thrombotic mediators. Resuscitative care is focused on optimizing cerebral perfusion and reducing secondary injury processes. Hypertonic saline is an effective osmotherapeutic agent for the treatment of intracranial hypertension and has immunomodulatory properties that may confer neuroprotection. This study examined the impact of hypertonic fluids on inflammatory/coagulation cascades in isolated head injury.

**Methods:**

Using a prospective, randomized controlled trial we investigated the impact of prehospital resuscitation of severe TBI (GCS < 8) patients using 7.5% hypertonic saline in combination with 6% dextran-70 (HSD) *vs *0.9% normal saline (NS), on selected cellular and soluble inflammatory/coagulation markers. Serial blood samples were drawn from 65 patients (30 HSD, 35 NS) at the time of hospital admission and at 12, 24, and 48-h post-resuscitation. Flow cytometry was used to analyze leukocyte cell-surface adhesion (CD62L, CD11b) and degranulation (CD63, CD66b) molecules. Circulating concentrations of soluble (s)L- and sE-selectins (sL-, sE-selectins), vascular and intercellular adhesion molecules (sVCAM-1, sICAM-1), pro/antiinflammatory cytokines [tumor necrosis factor (TNF)-α and interleukin (IL-10)], tissue factor (sTF), thrombomodulin (sTM) and D-dimers (D-D) were assessed by enzyme immunoassay. Twenty-five healthy subjects were studied as a control group.

**Results:**

TBI provoked marked alterations in a majority of the inflammatory/coagulation markers assessed in all patients. Relative to control, NS patients showed up to a 2-fold higher surface expression of CD62L, CD11b and CD66b on polymorphonuclear neutrophils (PMNs) and monocytes that persisted for 48-h. HSD blunted the expression of these cell-surface activation/adhesion molecules at all time-points to levels approaching control values. Admission concentrations of endothelial-derived sVCAM-1 and sE-selectin were generally reduced in HSD patients. Circulating sL-selectin levels were significantly elevated at 12 and 48, but not 24 h post-resuscitation with HSD. TNF-α and IL-10 levels were elevated above control throughout the study period in all patients, but were reduced in HSD patients. Plasma sTF and D-D levels were also significantly lower in HSD patients, whereas sTM levels remained at control levels.

**Conclusions:**

These findings support an important modulatory role of HSD resuscitation in attenuating the upregulation of leukocyte/endothelial cell proinflammatory/prothrombotic mediators, which may help ameliorate secondary brain injury after TBI.

**Trial registration:**

NCT00878631.

## Background

Traumatic brain injury (TBI) remains a leading cause of death and persistent neurocognitive impairment in civilian and military casualties [1]. The extent of brain damage is determined by the severity of primary mechanical injury and intensity of secondary biomolecular injury cascades causing neuroinflammation that contributes to cerebral edema, raised intracranial pressure and delayed cellular destruction [2]. TBI patients are also vulnerable to ensuing pathophysiological insults including hypotension and hypoxemia [3], initiating global ischemia/reperfusion and systemic immunoinflammatory responses that worsen neurological outcome and often lead to development of multiorgan dysfunction [4,5].

Prehospital care is a major determinant of long-term outcome in TBI patients. Current management involves expeditious infusion of crystalloids and colloids to limit secondary insults by hemodynamic stabilization and optimization of cerebral perfusion [6]. Yet, the ideal fluid for initial resuscitation of TBI remains unclear [7]; due to in part to a lack of adequately powered clinical trials comparing osmotic agents in neurotrauma [8]. Overzealous crystalloid resuscitation often serves to exacerbate delayed posttraumatic complications, including inflammatory-mediated cellular injury via enhanced cytokine release, endothelial activation, adhesion molecule expression and coagulopathy [9]. Conversely, resuscitation with hypertonic saline, alone or combined with dextran (HS/D), is emerging as effective first-line osmotherapeutic alternative for treatment of intracranial hypertension and cerebral edema [10,11]. Beyond its osmotic and hemodynamic properties, compelling laboratory and clinical data indicate that HS/D exerts immunomodulatory and antiinflammatory effects [12-15], including blunted cellular activation, cytokine production and adhesion molecule expression, that may also convey neuroprotection against brain secondary injury cascades [16,17].

It is increasingly evident that TBI elicits interdependent CNS and systemic inflammatory and coagulation cascades that contribute to secondary neuropathological sequelae [4,18]. These innate host defence responses to cerebral insults are characterized by rapid activation of resident microglia and astrocytes [19], brain microvascular ECs [20] and peripheral blood leukocytes (PMNs, monocytes) [21,22], with increased expression of multiple surface-active and soluble mediators, including inflammatory cytokines [23], adhesion molecules [24,25] and coagulation cofactors [18,26]. TNF-α is a central mediator of neuroinflammation after brain injury [19], inducing generalized endothelial activation/injury [27], direct neurotoxicity [28], reduced BBB integrity [23,29], and upregulated adhesion molecule expression [24,25,30], thus promoting leukocyte-EC interactions and cellular infiltration of injured brain parenchyma [31,32].

Based on the multi-step paradigm of leukocyte trafficking, inflammatory cells are recruited into the microvasculature via sequential adhesive interactions of selectins and integrins with their respective immunoglobulin family ligands [29]. Initial tethering and rolling of marginated cells along the vessel wall is mediated by L- (leukocyte; CD62L) and E- (endothelial; CD62E) selectins binding to sialyl-Lewis^X ^carbohydrate counter-receptors. Subsequent β_2_-integrin (CD11b)-dependent firm adhesion to endothelial ligands for intercellular and vascular cell adhesion molecules (ICAM-1, CD54; VCAM-1, CD106) allows leukocyte transmigration into damaged tissues [31]. Simultaneously, ligand-binding triggers leukocyte effector functions, such as respiratory burst and degranulation of azurophilic (CD63) and specific (CD66b) proteins [33]. Sequestration of activated leukocytes within the CNS inflicts secondary injury through generation of neurototoxic reactive species, granular proteases, and inflammatory cytokines, ultimately producing endothelial destruction, thrombosis and end-organ failure [34,35].

The vascular endothelium provides a critical interface for host inflammatory and coagulation responses to injury. Cytokine-activated or damaged ECs participate in hemostatic balance via synthesis of procoagulant TF and anticoagulant TM endothelial glycoproteins [36]. TBI patients are prone to coagulation abnormalities since the brain is rich in TF [26], which initiates the extrinsic pathway causing local and systemic hemostatic disturbances [37]. TNF-α is also a potent inducer of TF by ECs and monocytes [38,39], but inhibits cell-surface expression of TM [40], thus shifting the hemostatic balance towards intravascular thrombosis and hyperfibrinolysis with release of D-D fibrin degradation products [41]. Soluble isoforms of adhesion and coagulation molecules are also liberated during inflammation serving as circulating markers of endothelial activation or injury [42]. Collectively, these changes exemplify the molecular crosstalk between coagulation-inflammatory pathways as a key aspect in secondary brain injury progression [26,43].

In light of our previous findings in the same cohort of TBI patients [44] showing dramatically elevated S100B, NSE, and MBP brain-injury biomarkers in serum from patients resuscitated with NS relative to HSD-treated patients, along with the knowledge that these specific cellular injury markers are associated with disruption of the BBB and neuroinflammation [45,46], we hypothesized that prehospital resuscitation of head injury patients using 7.5% hypertonic saline with 6% dextran-70 (HSD) would attenuate the expression of selected cellular and soluble inflammatory/coagulation markers related to secondary brain injury cascades.

## Materials and methods

### Patients and Controls

Using as an *a priori *subgroup analysis within our previously reported, larger prospective randomized controlled trial to evaluate hypertonic fluid resuscitation [47], biomarkers of leukocyte and EC activation, inflammation and coagulation were studied in sixty-five severe TBI patients (Table 1). Patients were eligible for the study if at any time during prehospital care they experienced loss of consciousness due to isolated blunt head trauma and/or had a Glasgow Coma Scale (GCS) score of 8 or less. Patients were excluded if they had primary penetrating injury, had suffered severe life-threatening injury to organs other than the brain, received previous intravenous fluid therapy ≥ 50 mL, a time interval between arrival at scene and vascular access which exceeded 4 hours, were younger than 16 years, were pregnant, or had vital signs absent prior to randomization. The Phase II study was approved by the Research Ethics Boards of all participating agencies, along with the Canadian Therapeutic Products Directorate and registered at ClinicalTrials.gov (Identifier: NCT00878631). Prehospital informed consent was waived in accordance with the Tri-Council Policy Agreement for Research in Emergency Health Situations (Article 2.8). Patients were enrolled by advance life support or critical care paramedics, and informed consent was subsequently waived for blood sampling for this substudy. An age-matched (38.8 ± 8.5 yrs) control group of 25 asymptomatic adult donors, with no history of brain injury, were analyzed for determination of the selected cellular and soluble biomarkers after obtaining informed consent. Studies on human subjects were carried out according to the principles of the Declaration of Helsinki.

**Table 1:** Patient demographics and clinical characteristics on admission according to prehospital fluid resuscitation treatment groups.

*Variables*	Resuscitation Group		*P *Value^†^
		
	*HSD*	*NS*	
**Demographics**			
No. of Patients, *n*	30	35	
Age, yrs	41.8 ± 17.4	42.8 ± 18.8	0.85^a^
Sex Ratio (M/F)	19/11	25/10	0.79^c^
**Vital signs**			
GCS on Admission	5.6 ± 2.8	5.9 ± 2.6	0.53^b^
SBP, mm Hg	133.3 ± 16.2	130.8 ± 28.3	0.83^a^
ISS	34.9 ± 9.5	31.7 ± 14.6	0.19^b^
APACHE II	12.5 ± 5.9	14.8 ± 5.1	0.24^b^
SOFA	4.9 ± 1.7	4.9 ± 2.5	0.72^b^
MODS	3.8 ± 1.5	5.0 ± 3.3	0.64^b^
**Laboratory Values**			
Hb, g/dL	11.3 ± 1.6	11.9 ± 2.1	0.39^a^
Hct, %	32.9 ± 5.0	34.7 ± 5.9	0.40^a^
Plt, 10^9^/L	206.2 ± 41.5	219.5 ± 56.4	0.40^a^
pH	7.34 ± 0.06	7.33 ± 0.05	0.69^b^
BE, mEq/L	-2.1 ± 3.2	-2.4 ± 3.7	0.76^a^
aPTT, s	26.2 ± 5.3	24.4 ± 6.1	0.63^a^
INR	1.08 ± 0.21	1.12 ± 0.23	0.25^b^
Na, mmol/L	146.9 ± 4.5	143.5 ± 5.2	**0.03^a^**
Cl, mmol/L	115.4 ± 5.0	111.9 ± 7.0	0.07^a^
Osmolality, mOsm/kg	316.0 ± 20.1	306.7 ± 29.1	**0.05^a^**
**Fluids**			
Total fluids prehospital, L	0.4 ± 0.3	0.3 ± 0.1	0.83^a^
Total fluids in-hospital, L	5.4 ± 2.2	5.3 ± 2.5	0.96^a^
**Outcomes**			
GOS	3.7 ± 1.3	3.5 ± 1.5	0.81^b^
Length of stay, d	14.1 ± 13.6	14.7 ± 12.5	0.90^a^
Mechanical Ventilation, d	6.6 ± 5.2	9.1 ± 5.8	0.71^a^
Mortality, *n *(%)	4 (13.3)	6 (17.1)	0.67^c^

Abbreviations (normal ranges): NS, normal saline; HSD, hypertonic saline plus dextran; GCS, Glasgow Coma Scale; ISS, Injury Severity Score; APACHE II, Acute Physiologic and Chronic Health Evaluation; SOFA, Sequential Organ Failure Assessment; MODS, Multiple Organ Dysfunction Score; Hb, hemoglobin (12-18); Hct, hematocrit (37-52); Plt, platelets (130-400); aPTT, activated partial thromboplastin time (25-35 s); INR, International Normalized Ratio (0.8-1.2); BE, base excess (-2/+2); pH (7.35-7.45); GOS, Glasgow Outcome Scale score.

Unless otherwise stated, results are expressed as mean ± standard deviation (SD); ^†^statistical significance of differences between resuscitation treatment groups were assessed with ^a^*t*-test, ^b^Mann-Whitney *U*-test, or ^c^Chi-square test as appropriate, with significance set at *P *< .05.

### Study Design and Procedures

Eligible adult patients were randomly assigned to receive either a single prehospital bolus infusion of 250-mL of 7.5% hypertonic saline admixed with 6% dextran-70 (HSD; RescueFlow^® ^BioPhausia AB, Stockholm Sweden) as the experimental treatment, or 250-mL of the standard 0.9% normal saline (NS). Paramedics administered the treatment solutions as the initial resuscitation fluid given within 4 hours of the incident. After administration of the study fluid, all subsequent clinical treatment was performed according to best practice guidelines established by the American College of Surgeons Advanced Trauma Life Support (ATLS; http://www.facs.org/trauma/atls/edguidelines.html) and the Brain Trauma Foundation (BTF; http://www.braintrauma.org/site/PageServer?pagename=Guidelines). This included ongoing resuscitation by prehospital personnel with additional crystalloid as per existing protocols. At no point during initial or subsequent treatment was the standard-of-care withheld. Clinical data collected upon hospital admission included age, gender, mechanism of injury, and Injury Severity Score (ISS). Acute neurologic status was assessed by paramedics at the accident scene prior to resuscitation based on the patient's level of consciousness as categorized by GCS score. Motor response, verbal response and response to pain were noted, producing a total score from 3-15; with a range of 3-8 indicating severe injury. Severity of illness and organ dysfunctions was quantified and defined by Acute Physiologic and Chronic Health Evaluation (APACHE II), Sequential Organ Failure Assessment (SOFA) and Multiple Organ Dysfunction Score (MODS) at the time of admission to the ICU. In addition, neurological outcome at the time of hospital discharge (or ≤ 30 days) was assessed in consenting patients using the Glasgow Outcome Scale (GOS). The GOS is the most widely used method to describe overall outcome after head injury and is based upon the ability of recovering TBI patients to perform activities of daily living and the degree of assistance required. GOS was graded as follows: 1, indicates death; 2, persistent vegetative state; 3, severely disabled; 4, moderately disabled; 5, good recovery. GOS scores were further dichotomized into Good (GOS 4-5) or Poor (GOS 1-3) outcome, as previously described [44].

### Blood Sample Collection

Serial venous blood samples (totaling 25 mL) were drawn from each patient as soon as possible after admission to the emergency department and again at 12-, 24- and 48-h post-resuscitation. Specimens for cellular and soluble biomarker analyses were obtained from patients and controls using an evacuated tube collection system containing the appropriate anticoagulants or nonadditive tubes (Vacutainer, Becton Dickinson, Rutherford, NJ).

### Routine Clinical Hematology and Coagulation Analyses

Whole blood samples collected into trisodium citrate and K_2_EDTA tubes (Becton-Dickinson) at admission were analyzed for hematogical and hemostatic parameters using standard clinical chemistry techniques. Complete blood counts including hemoglobin (Hb), hematocrit (Hct), differential leukocyte and platelet (Plt) counts were performed using an automated Hematology Analyzer (Coulter A^C^T diff 2, Beckman-Coulter, Hialeah, FL). Prothrombin time (PT, s) and activated partial thromboplastin time (aPTT, s) were measured by a Blood Coagulation System; International Normalized Ratio (INR) was calculated and reported by the same instrument and was used as a surrogate for PT to account for institutional variability in this marker.

### Analyses of Plasma sTF, sTM, and D-D Concentrations

Trisodium citrate, sodium heparin, and K_2_EDTA anticoagulated blood samples were double centrifuged at 3000 ×*g *for 15 min to obtain platelet-poor plasma, which was stored in aliquots frozen at -80°C until analysis at a later date. Plasma concentrations of sTF, sTM and D-D were analyzed using commercially available IMUBIND quantitative ELISA kits according to the manufacturer's instructions (American Diagnostica Inc, Stamford, CI). Briefly, plasma samples for determination of sTF, sTM and D-D, respectively, were diluted 1:4, 1:5 and 1:50 with assay diluent; standards, samples, controls, and conjugate were incubated in precoated 96-well microplates. Any TF, TM and D-D present was sandwiched by the immobilized capture antibody and the enzyme-linked monoclonal antibody specific for these analytes. Following a wash to remove any unbound substances and/or antibody enzyme reagent, substrate solution was added to the wells allowing the colorometic reaction in proportion to the amount of sTF, sTM and D-D bound. The color development was stopped and the absorbance read at 450 nm with wavelength correction in an automated microplate photometer (EL340, BIO-TEK Instruments, Winooski, VT); protein concentrations in each sample were calculated according to the standard curve of mean optical densities of duplicate incubations. The lowest analytical detection limits for TF, TM, and DD are 10 pg/mL, 0.3 ng/mL and 2 ng/mL, respectively.

### Assessment of Cell-Surface Adhesion and Degranulation Molecules

Simultaneous multi-colour direct staining and flow cytometric analysis of granuloctye and monocyte cell membrane adhesion and degranulation molecules were performed using 100-μL aliquots of fresh untreated whole blood. Samples were pipetted directly into 12 × 75 mm polystyrene Falcon tubes and incubated with saturating concentrations of anti-CD14-APC, anti-CD62L-FITC, anti-CD11b-PE, anti-CD66b-FITC, and anti-CD63-PE surface stains (BD Biosciences, San José, CA) for 20 min at room temperature in the dark. Appropriate isotype-matched mAbs were added simultaneously to separate tubes as indicators of autofluorescence and non-specific antibody binding. Red blood cells were lysed by addition of 2 mL of 1 × FACS™ Lysing Solution for 10 min, and centrifuged at 500 ×*g *5 min at 20°C. The supernatant was washed with CellWASH™ and cell pellets resuspended in 400μ of 1% paraformaldehyde. Stained cell suspensions were acquired on a dual-laser FACSCalibur flow cytometer equipped with a 15-mW 488-nm air-cooled argon-ion laser supplemented with a 635-nm red diode laser (BD Biosciences). Instrument optical alignment, fluidics and day-to-day variability were monitored and adjusted using CaliBRITE^® ^fluorescence beads and FACSComp^® ^software. For each sample, 103 events were collected as list mode data at a flow rate of 500 events/sec using CellQuest^® ^software, with a live gate set using a bivariate dotplot of anti-CD14 reactivity vs. SSC orthogonal light-scatter profile to allow the distinction of monocytes and PMNs. Isotype-matched control samples confirmed specificity and served to define quadrant markers for two-dimensional dot-plot and fluorescence histogram analysis of positive and negative cell populations. Subsequent data analysis was performed using FlowJo software v.8.7 (Tree Star Inc., Ashland, OR). Results are expressed as the frequency of marker-positive events (% positive cells) and as indirect measures of cell-surface antigen density using linear mean fluorescence intensity (MFI).

### Determination of Serum Cytokines and Soluble Adhesion Molecule Concentrations

Blood samples collected into non-additive tubes were allowed to clot at room temperature and then centrifuged at 1000 ×*g *for 15 min. The serum supernatants were separated into 0.5-mL aliquots and frozen at -80°C until batch analysis. Samples were subsequently thawed immediately before analyses and assayed in duplicate for circulating concentrations of TNF-α, IL-10, sICAM-1, sVCAM-1, sE-selectin and sL-selectin, using commercially available quantitative ELISA kits according to the according to the manufacturer's protocol (Quantikine^®^, R&D systems Inc., Minneapolis MN). Absorbance was read at 450 nm with wavelength correction in an automated microplate photometer (EL340, BIO-TEK Instruments, Winooski, VT). Assay sensitivities for TNF-α and IL-10, corresponded to 0.12 and 0.5 pg/mL, respectively; sICAM-1, sVCAM-1, sE-selectin and sL-selectin had detection limits 0.096, 0.6, 0.009 and 0.3 ng/mL, respectively.

### Statistical Analysis

Baseline demographic and clinical characteristics are expressed as the mean ± standard deviation (SD). Data were assessed for normality and homogeneity of variance. Biomarker levels were treated as normally distributed continuous variables and expressed as mean ± standard error of the mean (SEM). Univariate and multivariate techniques were used according to the type of data being tested: intergroup comparisons between dichotomous variables including demographic and clinical characteristics were performed using Student's *t*-test for continuous variables and χ^2 ^test for categorical predictor variables; and the non-parametric Mann-Whitney *U *test for continuous variables that were not normally distributed. Serial comparisons (*time *× *treatment*) of biomarkers between the treatment groups and control group were made using repeated measures ANOVA with *post-hoc *Bonferroni/Dunn testing. All analyses were two-tailed, with a *p*-value < 0.05 indicating statistical significance.

## Results

### Patient Demographics and Clinical Characteristics

Table 1 summarizes demographic, injury and clinical characteristics of TBI patients enrolled into the substudy. The two fluid-treatment arms were well balanced with respect to age, GCS and other prognostic factors. There were no significant differences in presenting symptoms between the HSD and NS groups, with the exception of serum sodium and osmolality values, which were elevated in the HSD group.

### Circulating Leukocyte Counts

Total and differential leukocyte counts are shown in Table 2. Relative to normal control values both groups of resuscitated TBI patients exhibited a significant post-traumatic leukocytosis (up to 3-fold higher) upon hospital admission, which persisted throughout the 48-h observation period. Differential analyses revealed the generalized leukocytosis was comprised mainly of a profound neutrophilia (increased proportion and number) and a lesser monocytosis. Compared to patients treated with NS, HSD resuscitated patients showed significantly lower total white cell, PMN and monocyte counts on hospital admission, although beyond this time-point values were not significantly different between treatment groups.

**Table 2:** Absolute leukocyte counts and relative subset proportions in TBI patients and controls.

*Parameter*			*HSD*				*NS*		
		
	Control	Adm (≤ 3 h)	12 h	24 h	48 h	Adm (≤ 3 h)	12 h	24 h	48 h
**Leukocytes**									
Counts^1^	5.7 ± 0.1	13.0 ± 0.1* †	11.3 ± 0.3*	11.4 ± 0.6*	10.4 ± 0.2*	18.1 ± 0.8*	12.3 ± 0.6*	11.5 ± 0.2*	11.4 ± 0.5*
**PMNs**									
Counts	3.5 ± 0.1	10.8 ± 0.3* †	9.4 ± 0.1*	9.6 ± 0.2*	8.8 ± 0.1*	15.8 ± 0.4*	10.7 ± 0.2*	9.7 ± 0.1*	9.8 ± 0.1*
%	60.8 ± 0.6	82.4 ± 0.6*	83.5 ± 0.5*	83.5 ± 0.8*	83.9 ± 0.3*	86.5 ± 1.0*	86.3 ± 0.8*	84.1 ± 0.6*	85.4 ± 0.5*
**Monocytes**									
Counts	0.29 ± 0.01	0.54 ± 0.02* †	0.59 ± 0.03*	0.52 ± 0.02*	0.50 ± 0.04*	0.74 ± 0.04*	0.63 ± 0.02*	0.57 ± 0.03*	0.61 ± 0.01*
%	5.03 ± 0.13	4.37 ± 0.14	5.06 ± 0.13	4.64 ± 0.11	5.02 ± 0.12	4.14 ± 0.11	4.30 ± 0.12	5.27 ± 0.13	5.32 ± 0.11

^1^Mean ± standard error mean (SEM) cell concentrations (×10^9^/L).

*P < 0.05 *vs *age-matched healthy controls; ^†^P < 0.05 *vs *time-matched NS-treated patients by ANOVA

### Cell-Surface Adhesion and Degranulation Markers

**Figure 1:**
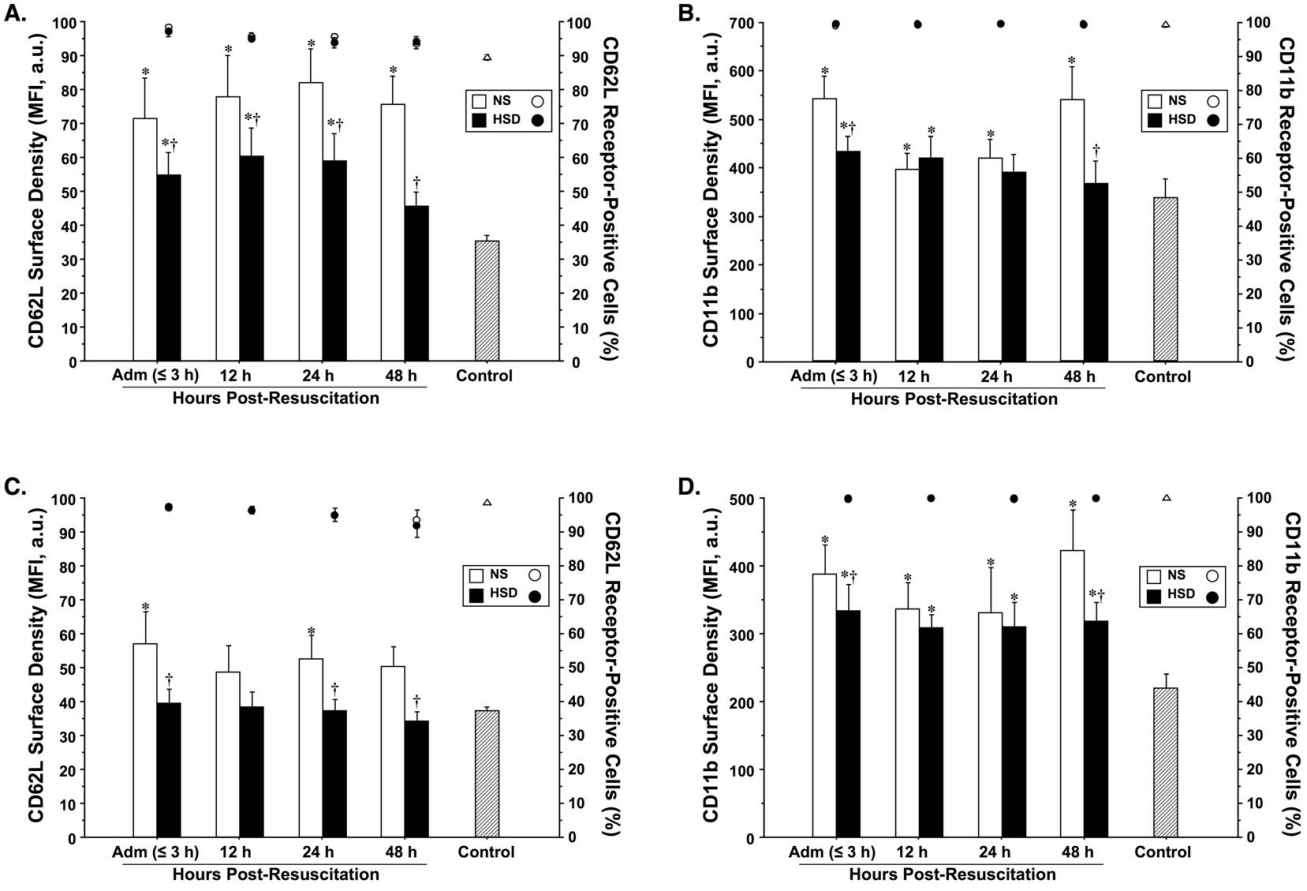
**Cell-surface expression of L-selectin (CD62L) and β_2_-integrin (CD11b) adhesion molecules on fresh peripheral blood monocytes (A-B) and polymorphonuclear neutrophils (PMNs; C-D) assessed by whole blood immunofluorescence flow cytometry**. Results were expressed as mean fluorescence intensity (MFI ± SEM; *bars*) in arbitrary units (a.u.) and the percentage of receptor-positive cells (*circles*), which correlate with antibody cell surface density. Blood was sampled serially from severe TBI patients resuscitated with normal saline (NS, *n *= 13) *vs *hypertonic saline-dextran (HSD, *n *= 10), upon hospital admission (Adm) and 12, 24, and 48-h post-resuscitation, and from healthy controls (*n *= 25). *P < 0.05 *vs *age-matched healthy controls (*n *= 25); ^†^*P *< 0.05 *vs *time-matched NS-treated patients by ANOVA.

Figure 1(A-D) shows the post-resuscitation time course of spontaneous CD62L and CD11b cell-surface adhesion molecule expression on fresh peripheral blood PMNs and monocytes, obtained from TBI patients and healthy blood donors. A majority (90-100%) of PMNs and monocytes from patients and controls were found to constitutively express CD62L and CD11b, with no significant temporal or intergroup differences in the proportion of cells bearing these receptors. Significantly higher surface density as quantified as mean fluorescence intensity (MFI) of CD62L and CD11b was found on both PMNs and monocytes from NS-treated patients on admission in comparison with HSD patients and controls. Expression of CD62L by PMNs and monocytes were elevated in the NS group across all sample times relative to control, whereas HSD treatment significantly down-regulated CD62L expression at all time points. CD11b expression on PMNs and monocytes exhibited a biphasic response pattern in the NS group, with an initial upregulation observed on admission, followed by a second peak at 48-h post-resuscitation. By contrast, after HSD, CD11b levels remained relatively constant over time at levels approaching control values.

**Figure 2:**
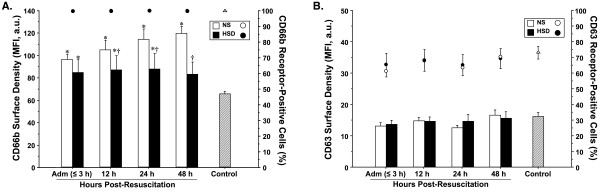
**Cell-surface expression of specific (CD66b; A) and azurophilic (CD63; **B**) granular proteins on fresh polymorphonuclear neutrophils (PMNs), assessed by whole blood immunofluorescence flow cytometry**. Results were expressed as mean linear fluorescence intensity (MFI ± SEM; *bars*) in arbitrary units (a.u.) and the percentage of receptor-positive cells (*circles*). Blood was sampled serially from severe TBI patients resuscitated with normal saline (NS, *n *= 13) *vs *hypertonic saline-dextran (HSD, *n *= 10), upon hospital admission (Adm) and 12, 24, and 48-h post-resuscitation, and from healthy controls (*n *= 25). *P < 0.05 *vs *age-matched healthy controls (*n *= 25); ^†^*P *< 0.05 *vs *time-matched NS-treated patients by ANOVA.

Figure 2(A-B) illustrates the temporal pattern of cell surface CD66b and CD63 degranulation marker expression on PMNs. Following resuscitation, both patient groups exhibited significant PMN degranulation, as evidenced by significantly higher surface expression of the specific granule marker CD66b in comparison to control. While the NS group showed progressively increasing levels of CD66b over time, HSD-treated patients displayed significantly lower CD66b protein density at all time points, which normalized by 48-h. Neither resuscitation strategy was found to influence PMN expression of CD63-positive azurophilic granules, since the surface density did not differ between patients and healthy controls at any time-point.

### Soluble Adhesion Molecules

**Figure 3:**
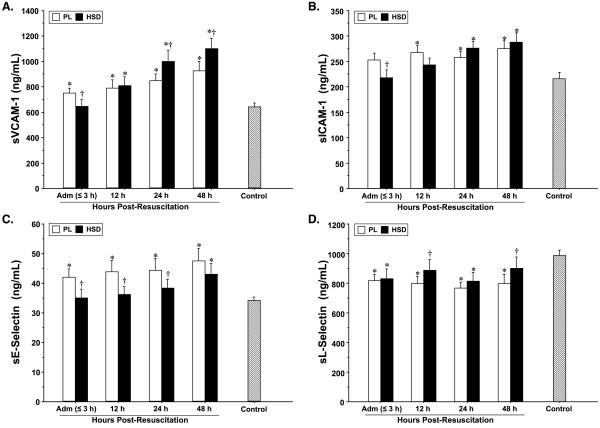
**Serum concentrations (mean ± SEM, ng/mL) of soluble vascular cell adhesion molecule (sVCAM-1), intercellular adhesion molecule (sICAM-1), endothelial selectin (sE-selectin) and leukocyte selectin (L-selectin) in severe TBI patients resuscitated with normal saline (NS, *n *= 35) or hypertonic saline-dextran (HSD, *n *= 30) and healthy controls (*n *= 25)**. Blood was sampled serially upon hospital admission (Adm) and 12, 24, and 48-h post-resuscitation. *P < 0.05 *vs *age-matched healthy controls (*n *= 25); ^† ^*P *< 0.05 *vs *time-matched NS-treated patients by ANOVA.

Serum concentrations (ng/mL) of the panel of four soluble adhesion molecules assayed in patients and healthy controls are shown in Figure 3(A-D). These soluble markers displayed unique kinetics of appearance in serum with significant differences observed between treatment groups. Concentrations of sVCAM-1 were significantly elevated upon admission in the NS group as compared to HSD-resuscitated patients and controls. sVCAM-1 levels continued to rise in all patients over time with peak values attained 48-h post-resuscitation in HSD patients. Similarly, admission concentrations of sICAM-1 were higher in the NS group as compared to HSD and also reached a maximum at 48-h. Comparatively, sE-selectin time-course values were markedly elevated in the NS group compared to HSD and control values. In contrast to the other measured soluble adhesion molecules, sL-selectin concentrations were significantly lower on admission in all patients in comparison with healthy controls. In HSD-resuscitated patients sL-selectin concentrations reached control values at 12 and 48 h, but levels remained depressed in the NS group throughout the observation period.

### Serum Cytokine Concentrations

**Figure 4:**
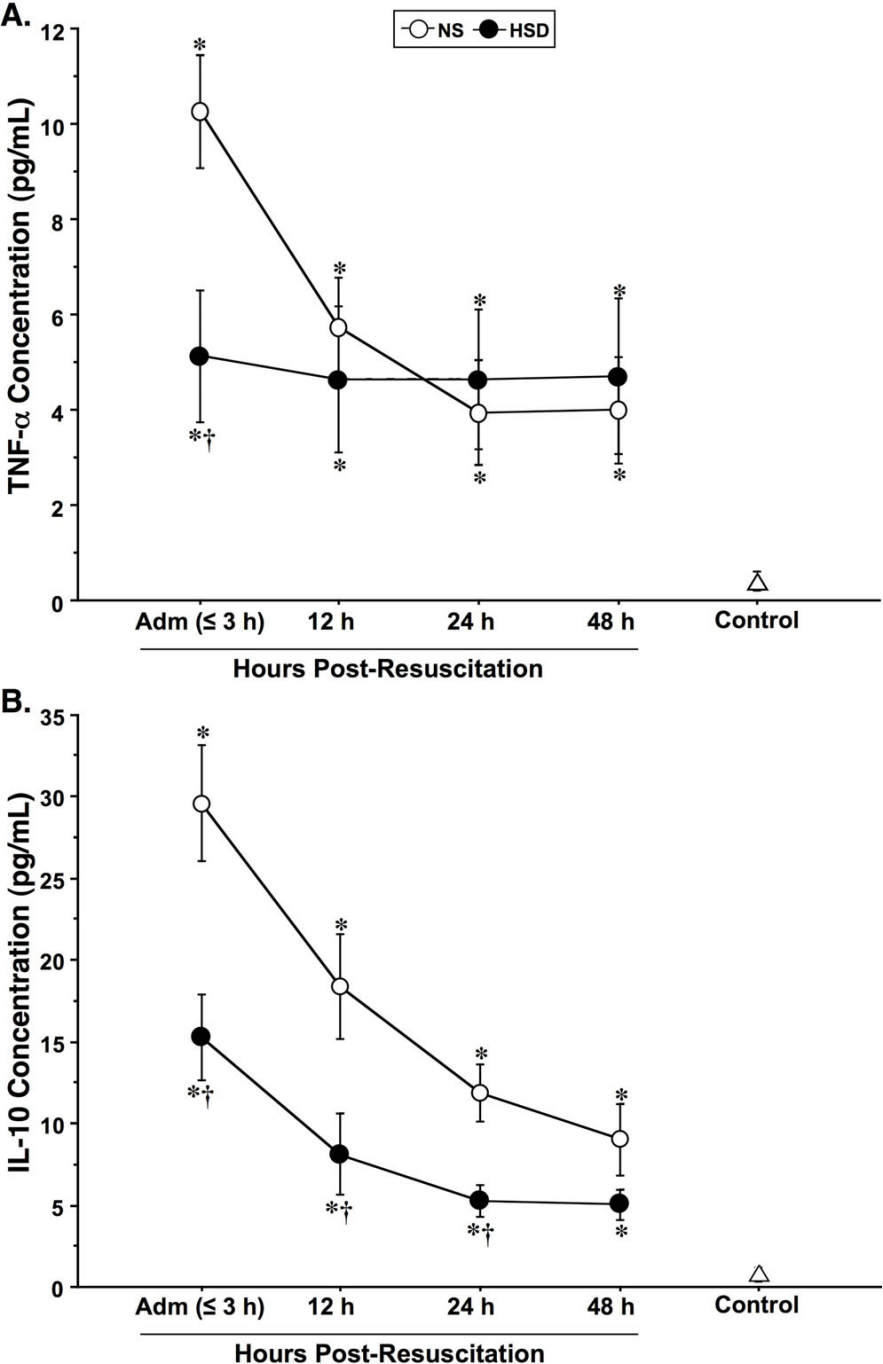
**Kinetics of serum concentrations (mean ± SEM, pg/mL) of pro- and anti-inflammatory cytokines TNF-α and IL-10 sampled serially in normal saline (NS, *n *= 35) and hypertonic saline-dextran (HSD, *n *= 30) resuscitated TBI patients, upon hospital admission (Adm), 12, 24, 48-h post-resuscitation**. *P < 0.05 *vs *age-matched healthy controls (*n *= 25); ^†^*P *< 0.05 *vs *time-matched NS-treated patients by ANOVA.

As shown in Figure 4 (A-B), the serum concentrations (pg/mL) of proinflammatory TNF-α and antiinflammatory IL-10 in resuscitated TBI patients were both markedly higher than those in control subjects. In particular, admission levels of TNF-α were 10-fold greater in NS-treated patients relative to control, and double those values observed in HSD patients. By comparison, TNF-α concentrations in HSD-treated patients did not show temporal variations but remained moderately above control values throughout the observation period. Peak serum IL-10 levels were 30-fold higher in the NS group on admission relative to control, and twice as high as HSD patients at all time points. IL-10 declined progressively over the observation period in both groups but remained above normal at 48 h.

### Plasma Coagulation Markers

**Figure 5:**
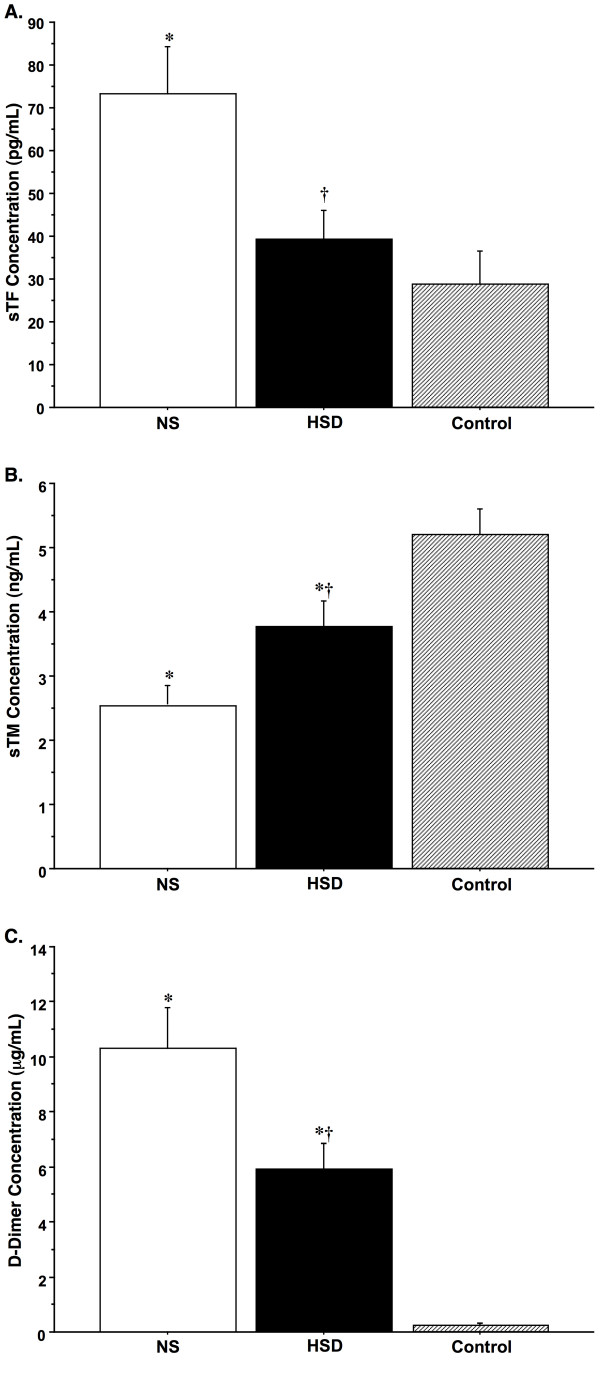
**Plasma concentrations of tissue factor (sTF; A), thrombomodulin (sTM; B) and D-dimers (D-D; C) in TBI patients resuscitated with normal saline (NS, *n *= 30) or hypertonic saline-dextran (HSD, *n *= 25) at the time of hospital admission**. *P < 0.05 *vs *age-matched healthy controls (*n *= 25); ^†^*P *< 0.05 *vs *time-matched NS-treated patients by ANOVA.

At the time of hospital admission, measurements of standard hemostasis indicators including INR, aPTT and platelet counts were within their normal ranges in both groups of TBI patients (Table 1); however, analyses of plasma concentrations of TF, TM, and D-D revealed pronounced differential effects of the two resuscitation strategies on patient coagulation profiles (Fig. 5A-C. Plasma concentrations of TF and D-D were significantly elevated in NS compared to HSD and control values. By contrast, HSD-resuscitated patients demonstrated significantly higher TM levels *versus *NS and control groups.

### Stratification of Biomarker Levels According to Dichotomized GOS

GOS scores at the time of hospital discharge/death within 30-days were dichotomized as Poor *versus *Good (i.e., GOS score of 1-3 *vs*. GOS score of 4-5, respectively). After dichotomization of the mean admission values for cellular and soluble inflammatory and coagulation markers were further stratified according to fluid resuscitation groups (Table 3). Overall, the marker levels were significantly higher in patients who had a poor outcome or died than in those who survived with good outcome, irrespective of fluid treatment. Further subgroup analysis revealed that HSD-resuscitated patients showing a good neurological outcome according to dichotomized GOS 4-5 scores exhibited the lowest levels of cellular and soluble markers. Conversely, the highest levels of most markers were observed in NS-resuscitated patients with poor outcome GOS 1-3. The notable exceptions to this pattern were sL-selectin and TM, which showed the lowest levels in those NS-treated patients with poor outcome.

**Table 3:** Admission levels of inflammatory and coagulation biomarkers in patients according to dichotomized Glasgow Outcome Scores (GOS) and resuscitation treatment group.

*Marker*		GOS Score		
	
	*HSD*		*NS*	
	*Poor *(1-3)	*Good *(4-5)	*Poor *(1-3)	*Good *(4-5)
**CD62L, MFI^§^**	48.5 ± 4.6^b^	33.7 ± 4.7^a,b^	58.5 ± 5.9	45.3 ± 4.4^a^
**CD11b, MFI^§^**	443.3 ± 58.7^b^	377.4 ± 28.3^a,b^	539.3 ± 91.8	505.9 ± 51.4
**CD66b, MFI^§^**	124.4 ± 28.4	74.6 ± 7.2^a,b^	135.1 ± 17.9	118.0 ± 8.3^a^
**sICAM-1, pg/mL**	228.3 ± 28.3^b^	211.1 ± 19.1^b^	254.4 ± 13.5	238.4 ± 29.0
**sVCAM-1, pg/mL**	713.8 ± 67.3^b^	574.2 ± 70.0^a,b^	757.0 ± 60.3	741.1 ± 54.8
**sE-Selectin, pg/mL**	33.9 ± 3.0^b^	31.1 ± 4.0^b^	45.2 ± 3.8	42.5 ± 4.6
**sL-Selectin, pg/mL**	849.2 ± 64.7^b^	875.2 ± 87.3^b^	770.3 ± 69.4	870.1 ± 60.0^a^
**TNF-α, pg/mL**	7.2 ± 2.4^b^	4.9 ± 1.7^a,b^	14.8 ± 1.4	6.9 ± 1.3^a^
**IL-10, pg/mL**	19.6 ± 6.3^b^	12.9 ± 2.2^a,b^	30.3 ± 4.9	26.4 ± 4.8^a^
**TF, pg/mL**	42.0 ± 4.3^b^	39.5 ± 12.6^b^	78.7 ± 10.5	65.8 ± 15.9^a^
**TM, ng/mL**	4.0 ± 1.1^b^	3.6 ± 1.3^b^	2.1 ± 1.3	2.8 ± 1.1
**D-D, μg/mL**	6.8 ± 2.3^b^	6.4 ± 1.2^b^	12.1 ± 2.1	7.6 ± 1.9^a^

Data are expressed as mean ± standard error mean (SEM) for each marker.

^§ ^MFI, mean fluorescence intensity of surface antigen expression by neutrophils.

Abbreviations: NS, normal saline; HSD, hypertonic saline-dextran; GOS, Glasgow Outcome Scale score.

^a^*P *< 0.05 *vs *GOS 1-3; ^b^*P *< 0.05 *vs *NS treated patients, by unpaired *t*-test.

## Discussion

It is generally accepted that severe head injury causes a profound inflammatory response within the brain leading to breakdown of the BBB with peripheral leukocyte invasion [5,48]. There is also abundant evidence for reciprocal alterations between CNS and peripheral inflammatory cells/mediators, which initiate systemic inflammatory cascades [4,49,50]. Growing recognition of the limitations of isotonic fluids for cerebral resuscitation has led to the search for alternative osmotic agents aimed at restoring cerebral perfusion and reducing intracranial pressure, while simultaneously conferring neutroprotection. In this randomized controlled trial, we investigated the potential immunomodulatory effects of prehospital HSD resuscitation on the production and interplay of leukocyte and endothelial adhesion molecules, cytokines, and coagulation cofactors in severe TBI patients. Although there is extensive preclinical and clinical experience evaluating the use of hypertonic fluids for their superior volume-expanding properties following extra-cranial insults, little is known concerning the potential impact of hyperosmolar therapy on acute immunoinflammatory response after isolated head injury [17,51]. HS/D has been shown to dampen posttraumatic hyperinflammatory responses and aberrant leukocyte-EC interactions, leading to reduced tissue cytotoxicity and end-organ damage in animal models of resuscitated hemorrhagic shock [52]; and recently the beneficial immunoregulatory properties of HS/D have also begun to translate into successful clinical trials of trauma patients [12,14]. However, to our knowledge, this is the first study to investigate the effects of HSD on the activation of cellular and molecular inflammatory/coagulation cascades initiated by TBI.

### Leukocyte and endothelial cell activation/adhesion molecules

Posttraumatic leukocyte adherence and activation are critical mediators of the pathogenesis of host tissue injury. In this study, we assessed the role of specific leukocyte and endothelial-derived activation/adhesion molecules in resuscitated TBI patients. Our results provide evidence that the expression of cell-associated and soluble adhesion molecules in severe TBI is differentially modulated depending on the type of fluid administered. After conventional crystalloid resuscitation, we found that relative to healthy controls, brain-injured patients exhibit extensive leukocyte mobilization, with significantly enhanced PMN and monocyte surface expression of L-selectin (CD62L) and β_2_-integrin (CD11b) adhesion molecules, along with exocytosis of the specific granular protein (CD66b). Along with increased expression of membrane-bound adhesion molecules, our analysis revealed that brain trauma caused elevated circulating levels of sE-selectin (sCD62E), sICAM-1 (sCD54) and sVCAM-1 (sCD106), but lower sL-selectin levels. A major finding of the present trial was that HSD markedly attenuated TBI-induced changes in these vital cell-associated and soluble molecules compared to NS.

The prominent neutrophilia and monocytosis observed in TBI patients is a well-known feature of acute inflammation that is consistently reported in patients sustaining polytrauma and closed head-injury [53]. This reflects the sympathetic hormonal surge following severe tissue damage, which massively mobilizes leukocytes into the circulation from the marginal pool in the microvasculature. As observed in the current study [14], we previously reported that early resuscitation with HSD greatly reduces shock-associated leukocyte redistribution via inhibition of catecholamine release. The ability of HSD to attenuate the magnitude of post-injury leukocytosis is clinically relevant since elevated leukocyte counts on hospital admission reliably correlate with adverse neurological outcome and mortality risk after TBI [54].

Leukocyte infiltration of the CNS is a hallmark of neuroinflammation and a key mechanism contributing to the neuropathogenesis of secondary brain injury [25,29]. Cerebral edema, raised intracranial pressure and neurotoxicity are mediated, at least in part, by intracerebral accumulation of blood-borne leukocytes [55,56]. Trafficking of inflammatory cells from the bloodstream into the brain parenchyma depends upon the regulated expression of paired adhesion molecules on the surface of infiltrating leukocytes and cerebrovascular ECs [29,30]. Results of this study show that leukocytes from TBI patients resuscitated with NS display rapid induction of constitutively expressed CD62L and activation of CD11b with translocation to the cell membrane from intracellular secretory granules [31]. Consistent with these findings, studies in humans and animals demonstrate upregulated cell-surface CD62L and CD11b expression on leukocytes following polytrauma and isolated head-injury [57]. CD11b expression is also closely associated with CD66b (co-localized in specific granules) and upon ligation forms a macromolecular complex that induces clustering of β_2_-integrins molecules, which potentiates avid binding to the endothelium with release of inflammatory mediators [58]. Importantly, the current results, showing that HSD prevents TBI-induced upregulation of leukocyte adhesion molecules, confirm previous *in vitro *human cellular studies [59,60], animal models [13,61,62] and clinical trials [12,14] of shock/resuscitation, demonstrating that hypertonicity inhibits leukocyte adherence and activation via downregulation of selectins, integrins and immunoglobulin molecules, thus rendering cells incapable of rolling or adhering to the endothelium.

Experimental studies show widespread microvascular endothelial activation [20,63] with upregulated adhesion molecule expression within 2-4 hours of neurological injury [24,64]. E-selectin is not normally present on non-inflamed cerebrovascular ECs, but is dramatically induced at the transcriptional level by inflammatory stimuli and upregulated expression mediates initial adherence of PMNs and monocytes to brain microvasculature after traumatic/ischemic insults [65]. Likewise, the constitutively low expression of ICAM-1 and VCAM-1 is rapidly upregulated on the cytokine-activated cerebrovascular ECs facilitating adhesion and transmigration of leukocytes into the CNS [35,66,67]. Immunohistochemical staining and microscopic analyses reveal early neutrophilic invasion of the brain parenchyma that peaks approximately 24 h after severe neurotrauma [68] and progresses to a predominance of monocyte/macrophage infiltrate by 72 h post-injury [69]. The temporal profile of cell recruitment in these earlier reports is consistent with the peak kinetics of CD62L and CD11b molecule expression we observed on blood PMNs and monocytes. Inappropriate sequestration of adherent leukocytes in postcapillary venules may aggravate neurovascular damage by further impairing cerebral blood flow [41] and by intracerebral release neurotoxic mediators [34].

The damaging role of infiltrating leukocytes in cerebral injury is substantiated by studies in which PMN depletion or administration of anti-adhesion antibodies [70] reversed impaired tissue perfusion, BBB dysfunction and reduced infarct size [30]. Furthermore, the extent of PMN accumulation is correlated with the degree of cerebral edema, injury severity, and poor outcome following TBI [21,55]. Although direct examination of cerebral leukocyte invasion was not possible in the present study, our recent findings in the same cohort of TBI patients showing marked elevations of serum S100B and NSE as indicators of brain damage after resuscitation with NS [44], are strongly suggestive of neuroinflammation, BBB impairment and cellular infiltration that may be ameliorated by HSD [45,46]. This assertion is also supported by intravital microscopy studies showing inhibition of leukocyte-EC interactions by hypertonic saline reduces microvascular permeability and tissue injury [13], and prevents BBB disruption allowing less intracerebral leukocyte sequestration in brain-injured animals compared to Ringers's [51,71].

Many adhesion molecules exist as both transmembrane proteins and biologically active soluble forms arising from proteolytic cleavage of the extracellular region of the membrane-bound molecule [42]. Increased levels of soluble adhesion molecules are reported in a variety of inflammatory conditions [72]. Although their physiological role(s) and clinical relevance is not fully resolved, it is generally accepted that elevated concentrations of these endothelial derivatives are liberated by activation-induced shedding, or arise as a result of direct vascular damage [73]. Our results showing enhanced serum concentrations of sE-selectin, sICAM-1 and sVCAM-1 are in accordance with the pattern of release of soluble selectins and immunoglobulin-type adhesion molecules characterized several experimental and clinical studies following brain injury [24,74,75]. High circulating concentrations of these molecules may serve as biomarkers for early diagnosis or prognosis of the development of the systemic inflammatory response syndrome, organ dysfunction and death after severe trauma or sepsis [76]. Similarly, increased sICAM-1 levels are reported to correlate with the extent of brain damage and breakdown of the BBB leading to poor outcomes after TBI [77]. Correspondingly, we found the highest levels of sE-selectin, sICAM and sVCAM in association with poor neurological outcome (i.e., GOS 1-3) in NS resuscitated patients. Notably, the only published study assessing the effects of hypertonic saline on the circulating profile of soluble adhesion molecules found lower sICAM-1 levels in the CSF of severe trauma patients compared to lactated Ringer's [78].

Soluble adhesion molecules are suggested to serve a functional role, either by inhibiting ongoing immunoinflammatory responses by competitive binding or conversely by inducing a response in ligand-bearing cells [72]. For instance, elevated sE-selectin and sICAM-1 have been shown to activate leukocytes following neurological insult [24], whereas high sL-selectin levels are closely associated with decreased cellular interactions and reduced microvascular damage in critically injured patients [79]. Our findings of initially low sL-selectin levels in TBI patients are consistent with earlier studies showing an inverse relationship between sL-selectin concentrations and increased risk of organ failure or death following isolated head injuries [80]. Furthermore, the current results showing reduced surface expression of CD62L in HSD-treated patients, accompanied by corresponding increases in sL-selectin and better patient outcome, are in accordance with our previous findings in shock patients [14], and supports the theory that sL-selectin shedding represents an endogenous regulatory mechanism to limit leukocyte-mediated injury [79].

### Inflammatory Cytokines

Cytokines are critical mediators of neuroinflammation after TBI [5,23], regulating a wide-variety of cellular functions through autocrine and paracrine signaling networks that initiate and perpetuate inflammatory reactions [25]. Severe TBI is associated with rapid and substantial increases in the synthesis and release of various pro/antiinflammatory cytokines into CSF and blood [4]. Experimental models of closed-head injury [30] and clinical studies [81,82] of TBI demonstrate early induction of TNF-α and IL-1β that peaks within 3-8 h of injury, followed by more sustained elevations of IL-6 and IL-10 [5,83]. In this study, we focused on the prototypical pro- and antiinflammatory cytokines, TNF-α and IL-10, respectively, since large increases in both correlate with head injury severity and are indicative of poor clinical outcome [84]. Like earlier experimental and clinical studies, our findings indicate peak serum concentrations of TNF-α and IL-10 are detectable within the first 3 h of injury, but remain above control values for up to 48 h. These cytokines are produced systemically and in high concentrations by resident microglia and infiltrating monocyte/macrophages in the acute phase of injury [22,69]. TNF-α induces capillary leak and edema formation [28], causing enhanced BBB permeability [29] and upregulated expression of adhesion molecules such as ICAM-1 and VCAM-1 on the surface of brain ECs and glial cells [24,85], exacerbating intracerebral leukocyte infiltration and microcirculatory dysfunction [23,41]. Inflammatory cytokines also stimulate astrocyte reactivity, contributing to increased neuroinflammation and development of secondary injury following neurotrauma [22,86]. Correspondingly, recent *in vivo *and *in vitro *animal studies have shown that both central and peripheral osmotic stimulation with hypertonic saline attenuates the brain's innate immune response to injury, reducing microglial activation, astrocytosis, cytokine production and associated neural tissue loss [15,16].

A remarkable finding of the current study was that HSD resuscitation halved the multifold increases in both TNF-α and IL-10 observed upon admission, but levels remained elevated for at least 48-h in comparison to control values. Although, this is the first published report of the effects of HSD on cytokine production after TBI, our results are consistent with several earlier studies in humans and animals demonstrating a more balanced pro versus antiinflammatory cytokine profile following hypertonic resuscitation of hemorrhagic shock or sepsis [52]. Previous studies examining TNF-α gene transcription and protein expression in response to hypertonicity have consistently shown inhibition in animal models of posttraumatic shock/resuscitation [87,88], *in vitro *exposure of human peripheral blood cells to hypertonic media [89-91], and *ex vivo *intracellular production by blood monocytes of resuscitated trauma patients [14]. Reports of endogenous IL-10 responses to hypertonicity are less consistent, exhibiting differential expression according to the dose administered, timing of treatment, type of injury, and tissue sample specificity. For example, we have demonstrated previously that in-hospital resuscitation with HSD enhances spontaneous and LPS-stimulated IL-10 expression in blood monocytes of shock patients [14], which corresponds with increased production by tissue macrophages in rodent models of shock/resuscitation [92-94]. By contrast, our present findings show that pre-hospital HSD treatment of TBI induced a roughly 50% reduction in circulating IL-10 levels lasting for at least 24 h, compared to NS. Similarly, clinically relevant doses of hypertonic saline *in vitro *were found to suppress IL-10 production in isolated LPS-stimulated human γδT cells [91] and to decrease circulating IL-10 concentrations in rats sustaining shock/resuscitation [9].

The ability of HSD to attenuate, but not abrogate, cytokine responses after TBI may be critical in light of their proposed duality in mediating both protective and injurious roles [27]. It has been suggested that high levels of TNF-α exert deleterious effects on the progression of tissue damage during the acute stages of CNS injury, but play a reparative role at lower levels [2]. Also, the relatively low IL-10 concentrations seen in HSD patients are consistent with the premise that moderate sustained levels of this potent antiinflammatory/immunosuppressive cytokine may be neuroprotective in early pathogenesis and subsequent termination of neuroinflammation, but detrimental at high levels [84,95]. In fact, it has been reported that up to 50% of isolated head-injury patients who survive an initial neurological insult subsequently die as a result of infection or non-neurological organ dysfunction [4]. Paralysis of cell-mediated immunity following severe TBI, partially induced by enhanced sympathetic-mediated IL-10 release, appears to be responsible for systemic immunosuppression with increased susceptibility to infectious complications [96]. This is consistent with our data showing that NS treated patients with poor outcome according to GOS score also exhibit the highest levels of TNF-α and IL-10. These findings support the idea that HSD leads to a more homeostatic cytokine profile that may alter secondary injury processes without compromising neurologic recovery.

### Coagulation Molecules

The posttraumatic inflammatory response impinges upon hemostatic regulatory mechanisms at multiple levels, including effects on procoagulant, anticoagulant and fibrinolytic systems [43]. Patients sustaining severe injury are at risk for coagulopathy due to concurrent hypothermia, blood loss, acidosis, and hemodilution [97]. Coagulation disorders are a frequent complication in patients with head injuries [26], which can be either hypercoagulable or hyperfibrinolytic and represent a powerful, independent predictor of prognosis [98]. TBI-associated hypercoagulability is associated with injury-mediated disruption of the cerebrovasculature with exposure of abundant subendothelial TF to circulating factor VII initiating the extrinsic pathway [18]. Intravenous fluids contribute to dilutional coagulopathy and also show intrinsic effects on the hemostatic system [99], but relatively few studies refer to the functional consequences after hypertonic resuscitation [100] and none evaluating the effects of HS/D on coagulation in TBI patients. Available *in vitro *and *in vivo *laboratory data suggest that hypertonic fluids exerts dose-dependent anticoagulant activity in animals or in serially diluted normal human plasma, as determined by routine clot-based assays and thromboelastography [101-104]. These studies provide evidence of direct anticoagulant effects of hypertonicity on plasma clotting factors and platelet activity and/or through interaction with inflammatory pathways [104,105].

Patients in the current study did not exhibit coagulopathy, as defined by standard clinical tests (i.e., INR > 1.3 or aPTT > 34 s), but significant differences were evident in circulating concentrations of coagulation cofactors between treatment groups. Based on the measured plasma sTF, sTM and D-D levels, our results show that compared to control, NS-resuscitated TBI patients exhibit activation of coagulation with concurrent down-regulation of anticoagulant systems and fibrinolysis. Furthermore, a key finding of this study was that HSD reduced TBI-induced increases in sTF and D-D levels by half, while maintaining sTM levels near control values. This suggests that hypertonic resuscitation may act to improve coagulofibrinolytic balance after TBI. Previous studies have shown liberation of a soluble TF from both vascular and extravascular sources following traumatic injury [36]. sTF is released into the circulation within hours of head injury [106], and inducible TF expression is rapidly upregulated on the surface of circulating monocytes after injury [38]. Although the source of the early increase in sTF is not clear, it likely the result of cleavage from TF-bearing monocytes and/or release from dysfunctional brain ECs in response to inflammatory cytokines [18,39]. Elevated systemic and intracerebral TNF-α release after TBI has been shown to have procoagulant effects, stimulating synthesis and release of sTF while suppressing cell-surface TM levels and the protein C pathway [35]. Normally, TM acts to control hemostasis through high-affinity binding of thrombin, which activates anticoagulant and antiinflammatory protein C pathways [40,97]. Elevated levels of sTM, as in the current study, are thought to reflect increased surface expression and shedding of transmembrane TM, which plays a role in regulating not only hemostasis, but also inflammation, thus providing a close link between these processes [40]. Although the biological relevance of changes in sTM in brain injury is not well characterized [35], emerging data from clinical and animal studies suggest that increases in endogenous sTM or exogenous administration of TM may have potent antithrombotic/antiinflammatory properties in inflammatory disease [107].

In conclusion, this study of severe head injury patients demonstrates that prehospital treatment with HSD attenuates inflammatory and coagulation cascades by modulating leukocyte and endothelial cell adhesion/activation molecule expression, pro/antiinflammatory cytokine release and pro/anticoagulant responses. These findings provide direct evidence that initial resuscitation with HSD imparts functional changes to inflammatory cells after TBI, which may inhibit the capacity for infiltration of damaged CNS tissues, thereby reducing potential neuroinflammatory events associated with secondary brain injury. Our results also suggest that by downregulating inflammatory mediator production, HSD may help prevent procoagulant TF-dependent derangements of hemostasis and fibrinolysis, which may otherwise contribute to intravascular thrombosis.

## Abbreviations

ANOVA: analysis of variance; BBB: blood-brain barrier; CNS: central nervous system; EC: endothelial cell; CSF: cerebrospinal fluid; D-D: d-dimers; HS/D: hypertonic saline with or without dextran; HSD: 7.5% hypertonic saline plus 6% dextran-70; ICAM-1: intercellular adhesion molecule-1; IL: interleukin; LPS: lipopolysaccharide; INR: international normalized ratio; MBP: myelin basic protein; MFI: mean fluorescence intensity; NSE: neuron specific enolase; PMN: polymorphonuclear neutrophil; PBS: phosphate-buffered saline; PT: prothrombin time; aPTT: activated partial thromboplastin time; TBI: traumatic brain injury; TF: tissue factor; TM: thrombomodulin; TNF-α: tumor necrosis factor alpha; VCAM-1: vascular cell adhesion molecule-1.

## Competing interests

The authors declare that they have no competing interests.

## Authors' contributions

Study Concept and Design: SBR, SGR, PNS, AJB, LJM. Study Implementation and Coordination: LJM, SBR, SGR, AJB. Data Collection and Analysis: SGR, NTC. Drafting Manuscript: SGR, NTC, SBR, SS. Revision of Manuscript: SGR, SBR, PNS, AJB, NTC, LJM, SC. All authors read and approved the final manuscript.
